# Do frailty screening tools assessed at emergency department presentation predict in-hospital mortality and length of stay in older adults with acute pulmonary edema?: A prospective cohort study

**DOI:** 10.1097/MD.0000000000048876

**Published:** 2026-05-15

**Authors:** Derya Dilaver, Görkem Alper Solakoğlu, Halil Emre Bilgiç, Uğur Durmuş

**Affiliations:** a Department of Emergency Medicine, İstanbul Medeniyet University, Prof. Dr. Süleyman Yalçin Göztepe City Hospital, Kadiköy, Istanbul, Turkey.

**Keywords:** acute heart failure, acute pulmonary edema, emergency department, frailty, in-hospital mortality, length of stay, older adults

## Abstract

Frailty is common in older adults with acute heart failure, yet whether bedside frailty instruments assessed during acute physiological decompensation can predict short-term hospital outcomes remain uncertain. This study examined the association between frailty, measured at emergency department presentation, and in-hospital mortality and length of stay in older patients hospitalized with acute pulmonary edema. A prospective cohort study was conducted at a tertiary emergency department between January 15 and September 15, 2025. Consecutive adults aged ≥ 65 years who presented with acute pulmonary edema and required hospitalization were enrolled. Frailty was assessed at the time of emergency department presentation using the Clinical Frailty Scale (CFS), Programme de Recherche sur l’Intégration des Services de Maintien de l’Autonomie-7 (PRISMA-7), and the Tilburg frailty indicator (TFI). Primary outcomes were in-hospital mortality and prolonged hospitalization, defined as a length of stay exceeding the 75th percentile of the cohort’s distribution (>12 days). Multivariable logistic regression and receiver operating characteristic analysis were used. Among 253 participants, frailty prevalence was high across instruments (71–73%). Median hospital length of stay was 8 days (interquartile range 5–12), and in-hospital mortality was 6.7% (n = 17). In adjusted analyses, none of the frailty instruments were independently associated with in-hospital mortality or prolonged hospitalization. For mortality, no baseline variable reached statistical significance; CRP showed a trend (OR 1.01; 95% CI: 1.00–1.01; *P* = .073). The history of coronary artery disease was independently associated with lower odds of prolonged hospitalization (OR 0.52; 95% CI: 0.28–0.98; *P* = .042). All frailty instruments showed poor discrimination for both outcomes, with AUC values ranging from 0.51 to 0.62 and confidence intervals that included 0.50. Older patients hospitalized with acute pulmonary edema had a high prevalence of frailty, yet frailty instruments assessed at emergency department presentations showed limited ability to predict short-term in-hospital outcomes. Frailty scoring performed during an acute decompensation episode may not reliably reflect baseline vulnerability; its role in this setting may be better suited to care planning and post-discharge risk stratification than to short-term prognostication.

## 1. Introduction

Heart failure (HF) affects over 64 million individuals worldwide.^[1]^ Acute heart failure (AHF) is a clinical condition in which HF syndrome presents either as a new-onset disease or a rapid or gradual worsening of preexisting chronic HF, characterized by symptoms and/or signs severe enough to require urgent medical evaluation and typically requiring an emergency department visit or unplanned hospitalization. In current acute care practice, particularly among adults aged ≥65 years, AHF is associated with a high risk of in-hospital mortality.^[2,3]^

Frailty is common in older adults and is associated with adverse outcomes such as hospitalization, falls, morbidity, and mortality.^[4,5]^ Although cardiovascular diseases can accelerate the frailty process, frailty can also adversely affect the outcomes of cardiovascular diseases.^[6]^ Observational evidence supports the clinical importance of frailty in patients with HF regarding hospital-centered outcomes. In cardiovascular disease, frailty facilitates the prediction of mortality, independent of comorbidity burden, age, and disease severity.^[7]^ A 2025 systematic review showed that frail patients with HF have a longer hospital length of stay than non-frail patients, highlighting the practical impact of frailty on bed utilization and the course of recovery.^[8]^ An important distinction exists, however, between baseline frailty – reflecting a patient’s habitual functional reserve – and acute functional impairment arising from decompensation. Acute heart failure hospitalizations are associated with profound inactivity and an acute rise in inflammatory mediators that may exacerbate functional decline beyond the patient’s premorbid trajectory, making it difficult to distinguish true frailty from reversible acute deterioration when assessment occurs at the peak of illness.^[9]^

Although the clinical relevance of frailty is increasingly documented in both HF management and geriatric emergency medicine, the current literature highlights ongoing uncertainty regarding which frailty tool best predicts emergency department and hospital outcomes.^[10]^ Numerous tools are available, each emphasizing various aspects of vulnerability. For instance, the Clinical Frailty Scale (CFS), which reflects patients’ current functional and physical status, is a practical instrument for rapid clinical stratification. Its feasibility and reliability have been demonstrated in emergency department studies.^[11]^ PRISMA-7 is a brief questionnaire that can be completed quickly, without specialized training, and it offers diagnostic utility for screening frailty. Associations with adverse outcomes with this tool have been reported across different healthcare settings.^[12]^ The Tilburg frailty indicator (TFI) is a multidomain instrument covering the physical, psychological, and social aspects of frailty, and its performance, particularly in acute care, requires further evaluation across different clinical contexts.^[13]^

Although the literature includes many studies on the impact of frailty on outcomes in chronic HF, prospective studies comparing the performance of different frailty instruments in AHF are limited. A further unresolved question concerns the timing of assessment: frailty instruments applied during an acute decompensation episode may capture transient functional decline rather than the patient’s true premorbid baseline, as the correct timing for diagnosing frailty in heart failure has not yet been established and acute illness may confound scores on function-based instruments.^[14]^ We hypothesized that frailty assessed at emergency department presentations would show limited independent association with short-term inhospital outcomes – specifically in-hospital mortality and prolonged hospitalization – owing to the potential overestimation of frailty severity during acute physiological decompensation. This study therefore aimed to determine the association between frailty, measured at presentation, and in-hospital outcomes in older patients hospitalized with acute pulmonary edema, and to assess the discriminative performance of 3 commonly used frailty instruments for these outcomes.

## 2. Materials and methods

The study was conducted at the Department of Emergency Medicine at a Tertiary hospital between January 15, 2025 and September 15, 2025. This was a prospective cohort study, and frailty instruments were administered as structured questionnaires. Ethical approval was obtained from the Istanbul Medeniyet University Faculty of Medicine Non-Interventional Health Research Ethics Committee (decision no. 2025/01-10). This study was conducted in accordance with the principles of the Declaration of Helsinki.

### 2.1. Study setting and sample

Göztepe Prof. Dr. Süleyman Yalçin City Hospital is a tertiary training and research hospital affiliated with Istanbul Medeniyet University, with approximately 4,00,000 adult emergency department (ED) visits annually. Eligible participants were adults aged ≥ 65 years who presented to the Department of Emergency Medicine at Göztepe Prof. Dr. Süleyman Yalçin City Hospital with acute pulmonary edema and required hospitalization. Between the specified dates, 288 consecutive patients aged ≥ 65 years who presented to the emergency department with acute pulmonary edema and required hospitalization were screened for inclusion. Fifteen patients were excluded due to endotracheal intubation, 18 due to altered mental status, and 2 due to loss of follow-up. Finally, 253 patients were included in the analysis. In this study, patients’ demographic characteristics and history of chronic diseases were recorded. Frailty was assessed using the CFS, PRISMA-7, and TFI at the time of emergency department presentation, based on each patient’s clinical status at evaluation. The laboratory parameters included complete blood count, creatinine, troponin, sodium, and C-reactive protein (CRP) levels. Vital signs and echocardiographic findings were also documented. Clinical outcomes were evaluated in terms of length of hospital stay (days) and mortality status. Prolonged hospitalization was defined as a length of stay exceeding the 75th percentile of the cohort’s observed distribution, corresponding to >12 days. This upper-quartile threshold approach has been applied in prior heart failure studies to identify patients whose hospital course deviated from the typical inpatient trajectory.^[15,16]^ The characteristics of the 3 frailty scores used in this study are described below. Heart failure was categorized according to left ventricular ejection fraction into HFpEF, HFrEF, and HFmrEF.

### 2.2. Clinical Frailty Scale (CFS)

Clinical Frailty Scale was developed based on its application in 2035 participants enrolled in the Canadian Study of Health and Aging. On this scale, a score of 1 indicates a very fit individual, whereas a score of 9 indicates the most severe level of frailty.^[17]^

### 2.3. Tilburg frailty indicator

This scale, which has a Turkish validity and reliability study, assesses physical, psychological, and social frailty. It includes 15 questions covering physical, psychological, and social domains. Patients with a score of 5 or higher are considered frail.^[18]^

### 2.4. PRISMA-7 questionnaire

PRISMA-7 is a 7-item yes/no questionnaire developed by Raîche et al in 2007; individuals scoring ≥ 3 is classified as frail. The items included being older than 85 years, sex, the presence of a health problem that limits daily activities, and the need for social and physical support.^[19]^

### 2.5. Study size

The study enrolled all consecutive eligible patients presenting during the prespecified 8-month period. No formal sample size calculation was performed prior to data collection, as this was an exploratory prospective cohort study aimed at describing frailty prevalence and its association with in-hospital outcomes in a real-world emergency department population. The resulting sample of 253 patients provided adequate representation of the target population within the study setting; the absence of a power calculation is acknowledged as a methodological limitation.

### 2.6. Bias

Three potential sources of bias were identified. First, patients with endotracheal intubation or altered mental status were excluded from enrollment, as frailty assessment required patient cooperation. This exclusion criterion may have led to underrepresentation of the most severely ill individuals, potentially attenuating the observed association between frailty and adverse outcomes (selection bias toward less severe illness).

Second, frailty was assessed at the time of emergency department presentation rather than reflecting premorbid baseline status. Acute physiological derangement associated with pulmonary edema may transiently worsen functional performance and inflate scores on function-based instruments, resulting in overclassification of frailty in a subset of patients (misclassification bias toward frailty overestimation).

Third, the absence of acute illness severity markers – including natriuretic peptide levels, intensive care unit admission, and the need for noninvasive ventilation – limits the degree of confounder adjustment achievable in the multivariable models (residual confounding).

### 2.7. Statistical analysis

Categorical variables were expressed as frequencies and percentages. For between-group comparisons, the chi-square test was used for categorical variables, and Fisher exact test was used when appropriate. Continuous variables were first assessed for normality using the Shapiro–Wilk test. Normally distributed variables are reported as mean ± standard deviation, whereas non-normally distributed variables are reported as median with the 25th to 75th percentiles. Student *t* test was used to compare normally distributed continuous variables, and the Mann–Whitney *U* test was used for non-normally distributed continuous variables. Variables with a *P* value below .10 in univariate analyses, together with those judged a priori to be clinically relevant based on existing literature on acute heart failure and frailty, were candidates for inclusion in the multivariable logistic regression models. Separate models were constructed for each outcome: in-hospital mortality and prolonged hospitalization. Each model included the 3 frailty instruments alongside clinical and laboratory covariates selected by the above criteria. Length of hospital stay was treated exclusively as an outcome variable and was not entered as a covariate in the mortality model, as it represents a post-admission result rather than a baseline predictor. The goodness-of-fit of each model was evaluated using the Hosmer–Lemeshow test. Receiver operating characteristic (ROC) analysis was performed to assess the discriminative ability of continuous variables independently associated with each outcome and the area under the curve (AUC) was calculated with 95% confidence intervals.

All 253 patients included in the final analysis had complete data for the variables reported in this study, with the exception of 1 patient who had a missing CRP value and was therefore excluded from logistic regression analyses (n = 252 for both regression models). No imputation was performed.

Statistical analyses were conducted using SPSS version 26 (IBM Corp., released 2019; IBM SPSS Statistics for Windows, Version 26.0; Armonk: IBM Corp.). Statistical significance was set at *P* < .05 for all comparisons.

## 3. Results

A total of 253 patients were included in this study. Of the participants, 63.6% were aged 65 to 84 years (n = 161) and 36.4% were aged ≥ 85 years (n = 92). The sex distribution was 45.1% male (n = 114) and 54.9% female (n = 139) patients. On presentation, the median systolic blood pressure was 135 mm Hg (117–161), and the median diastolic blood pressure was 74 mm Hg (64–83). The comorbidity burden was substantial, with 94.1% of patients having at least 1 comorbidity (n = 238). The most common comorbidities were hypertension (78.7%), diabetes mellitus (49.0%), coronary artery disease (46.2%), and HF (36.0%), and chronic obstructive pulmonary disease and chronic kidney disease were reported in 13.8% and 23.7% of patients, respectively. Laboratory findings showed a median CRP level of 16 mg/L (7–48), anemia prevalence of 76.3% (n = 193), elevated troponin in 33.2% (n = 84), and hyponatremia in 20.9% (n = 53) of patients.

The frailty indices were high: median CFS was 5 (4–5.5), PRISMA-7 was 3 (2–4), and TFI was 5 (4–7). The prevalence of frailty according to the standard cutoffs was 73.1% for CFS ≥ 5, 72.3% for PRISMA-7 ≥ 3, and 71.1% for TFI ≥ 5. The median hospital length of stay was 8 days,^[5-12]^ and the overall in-hospital mortality rate was 6.7% (n = 17).

Among patients who died, the median length of stay was 10 days (IQR: 7–22) versus 8 days (IQR: 5–12) among survivors. Although the mortality group had a longer stay, the difference was not statistically significant (*P* = .072). Mortality occurred in 6/58 (10.3%) patients with prolonged hospitalization and 11/195 (5.6%) patients with shorter stays. Although mortality appeared higher in the prolonged stay group, this difference was not statistically significant (*P* = .233). The associations between clinical characteristics, mortality, and hospital length of stay are presented in Table 1.

**Table 1:** Clinical, laboratory, and frailty variables associated with prolonged length of stay and mortality: bivariate group comparisons.

Variable	Prolonged hospital stay (n = 58)	Short hospital stay (n = 195)	*P*	Mortality (n = 17)	No mortality (n = 236)	*P*
Age						
65–84	41 (70.7)	120 (61.5)	.203	9 (52.9)	152 (64.4)	.343
≥85	17 (29.3)	75 (38.5)	.203	8 (47.1)	84 (35.6)	.343
Male sex	26 (44.8)	88 (45.1)	.968	8 (47.1)	106 (44.9)	.864
Systolic blood pressure, mm Hg (median [IQR])	133 (114–167)	136 (118–160)	.985	132 (112–154)	136 (117–162)	.517
Diastolic blood pressure, mm Hg (median [IQR])	74 (64–88)	74 (65–82)	.880	70 (60–80)	74 (65–84)	.218
COPD	10 (17.2)	25 (12.8)	.392	3 (17.6)	32 (13.6)	.713
CHF	17 (29.3)	74 (37.9)	.229	7 (41.2)	84 (35.6)	.643
Hypertension	46 (79.3)	153 (78.5)	.890	14 (82.4)	185 (78.4)	.999
Diabetes mellitus	32 (55.8)	92 (47.2)	.285	9 (52.9)	115 (48.7)	.737
Coronary artery disease	20 (34.5)	97 (49.7)	.041*	5 (29.4)	112 (47.5)	.149
Chronic kidney disease	16 (27.6)	44 (22.6)	.430	1 (5.9)	59 (25)	.114
Any comorbidity present	55 (94.8)	183 (93.8)	.999	16 (94.1)	222 (94.1)	.999
CRP, mg/L (median [IQR])	20 (9–87)	15 (6–43)	.054	22 (13–70)	15 (6–46)	.076
Anemia	44 (75.9)	149 (76.4)	.931	16 (94.1)	177 (75)	.082
Acute kidney injury on admission†	13 (22.4)	29 (14.9)	.333	3 (17.6)	39 (16.5)	.339
Chronic kidney disfunction on admission†	12 (20.7)	37 (19)	.333	1 (5.9)	48 (20.3)	.339
Troponin elevation	21 (36.2)	63 (32.3)	.580	6 (35.3)	78 (33.1)	.850
Hyponatremia	12 (20.7)	41 (21)	.956	6 (35.3)	47 (19.9)	.134
Heart failure phenotype by most recent EF, %			.827			.635
HFpEF	33 (56.9)	102 (52.3)	–	9 (52.9)	126 (53.4)	–
HFrEF	19 (32.8)	71 (36.4)	–	5 (29.4)	85 (36)	–
HFmrEF	6 (10.3)	22 (11.3)	–	3 (17.6)	25 (10.6)	–
Frailty indices						
CFS score ≥ 5	44 (75.9)	141 (72.3)	.592	14 (82.4)	171 (72.5)	.572
PRISMA-7 score ≥ 3	43 (74.1)	140 (71.8)	.726	15 (88.2)	168 (71.2)	.166
TFI score ≥ 5	40 (69)	140 (71.8)	.676	15 (88.2)	165 (69.9)	.164

CFS = Clinical Frailty Scale, CHF = congestive heart failure, CKD = chronic kidney disease, COPD = chronic obstructive pulmonary disease, CRP = C-reactive protein, EF = ejection fraction, HFmrEF = heart failure with mildly reduced ejection fraction, HFpEF = heart failure with preserved ejection fraction, HFrEF = heart failure with reduced ejection fraction, IQR = interquartile range, PRISMA-7 = Programme de Recherche sur l’Intégration des Services de Maintien de l’Autonomie-7, TFI = Tilburg frailty indicator.

* Indicates statistical significance at *P* < .05.

† Acute kidney injury and chronic kidney dysfunction on admission were defined based on serum creatinine levels at presentation; chronic kidney disease refers to a preexisting diagnosis documented in the patient’s medical history.

In the multivariable logistic regression for in-hospital mortality, none of the included variables reached statistical significance at *P* < .05. CRP showed a trend toward association with mortality (OR 1.01; 95% CI: 1.00–1.01; *P* = .073), and chronic kidney disease trended toward a protective association (OR 0.16; 95% CI: 0.02–1.25; *P* = .081). None of the frailty instruments were independently associated with in-hospital mortality (all *P* > .25; Table 2). In the multivariable analysis for prolonged hospitalization, a history of coronary artery disease was independently associated with lower odds of prolonged hospitalization (OR 0.52; 95% CI: 0.28–0.98; *P* = .042), indicating that patients with established coronary artery disease were less likely to experience a prolonged inpatient course. CRP did not reach significance (OR 1.00; *P* = .121), and none of the frailty instruments were independently associated with prolonged hospitalization (all *P* > .35; Table 3).

**Table 2:** Multivariable logistic regression analysis for inhospital mortality (n = 252).

Variable	Odds ratio	95% confidence interval	*P*
History of chronic kidney disease	0.16	0.02–1.25	.081
Anemia	4.65	0.59–36.57	.144
CRP, mg/L	1.01	1.00–1.01	.073
CFS score ≥ 5	0.64	0.10–4.08	.636
PRISMA-7 score ≥ 3	1.69	0.18–15.65	.646
TFI score ≥ 5	3.12	0.41–23.60	.270

Length of hospital stay was excluded from this model as it represents a post-admission outcome rather than a baseline predictor.

CFS = Clinical Frailty Scale, CRP = C-reactive protein, PRISMA-7 = Programme de Recherche sur l’Intégration des Services de Maintien de l’Autonomie-7, TFI = Tilburg frailty indicator.

**Table 3:** Multivariable logistic regression analysis for prolonged hospitalization (n = 252).

Variable	Odds ratio	95% confidence interval	*P*
History of coronary artery disease	**0.52**	**0.28–0.98**	**.042**
CRP, mg/L	1.00	1.00–1.01	.121
CFS score ≥ 5	1.63	0.58–4.53	.353
PRISMA-7 score ≥ 3	1.08	0.37–3.17	.885
TFI score ≥ 5	0.59	0.23–1.52	.273

Bold indicates statistical significance at *P* < .05.

CFS = Clinical Frailty Scale, CRP = C-reactive protein, PRISMA-7 = Programme de Recherche sur l’Intégration des Services de Maintien de l’Autonomie-7, TFI = Tilburg frailty indicator.

**Figure 1.**
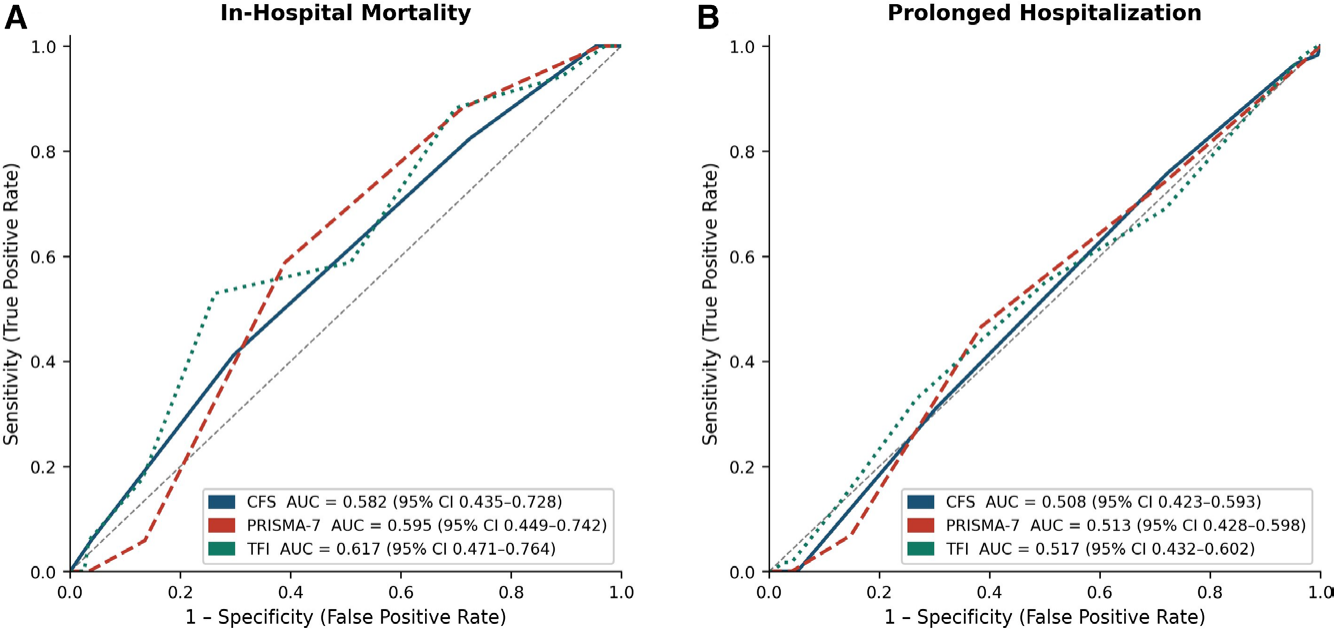
Receiver operating characteristic curves for the 3 frailty instruments in predicting in-hospital mortality (A) and prolonged hospitalization (B). AUC values with 95% confidence intervals are shown for each instrument. AUC = area under the curve, CFS = Clinical Frailty Scale, CI = confidence interval, PRISMA-7 = Programme de Recherche sur l’Intégration des Services de Maintien de l’Autonomie-7, TFI = Tilburg frailty indicator.

ROC analysis showed poor discriminative ability for all 3 frailty instruments across both outcomes (Table 4). For in-hospital mortality, AUC values were 0.582 (95% CI: 0.435–0.728) for CFS, 0.595 (95% CI: 0.449–0.742) for PRISMA-7, and 0.617 (95% CI: 0.471–0.764) for TFI. For prolonged hospitalization, AUC values were 0.508 (95% CI: 0.423–0.593) for CFS, 0.513 (95% CI: 0.428–0.598) for PRISMA-7, and 0.517 (95% CI: 0.432–0.602) for TFI. The 95% confidence intervals for all AUC estimates included 0.50, indicating that none of the frailty instruments performed better than chance for either outcome (Fig. 1).

**Table 4:** Discriminative performance of frailty instruments: receiver operating characteristic analysis.

Frailty instrument	Outcome	AUC	95% confidence interval
CFS	In-hospital mortality	0.582	0.435–0.728
PRISMA-7	In-hospital mortality	0.595	0.449–0.742
TFI	In-hospital mortality	0.617	0.471–0.764
CFS	Prolonged hospitalization	0.508	0.423–0.593
PRISMA-7	Prolonged hospitalization	0.513	0.428–0.598
TFI	Prolonged hospitalization	0.517	0.432–0.602

AUC values ≤ 0.70 with confidence intervals crossing 0.50 indicate poor discrimination.

AUC = area under the curve, CFS = Clinical Frailty Scale, PRISMA-7 = Programme de Recherche sur l’Intégration des Services de Maintien de l’Autonomie-7, TFI = Tilburg frailty indicator.

## 4. Discussion

This study examined the association between frailty and in-hospital mortality, and length of hospital stay among older patients admitted to the emergency department with acute pulmonary edema. Although the prevalence of frailty in the cohort was high, none of the 3 frailty instruments were independently associated with either outcome in adjusted analyses. After removing length of hospital stay from the mortality model – as it represents a post-admission result rather than a baseline predictor – no variable reached statistical significance, and all frailty instruments showed poor discriminative ability with AUC values below 0.62 and confidence intervals that included 0.50. For prolonged hospitalization, a history of coronary artery disease (CAD) was the only independent predictor, and it was associated with lower odds of prolonged stay (OR 0.52; 95% CI: 0.28–0.98; *P = *.042). This finding suggests that, in the acute care context, the effect of frailty on in-hospital adverse events may be masked by the burden of complications during admission, which may lead to an increased length of stay. A recent systematic review and meta-analysis found an association between frailty and longer LOS, noting that multiple frailty instruments were used across studies and that frailty often coexisted with multiple comorbidities.^[8]^

The observation that a history of CAD was associated with lower odds of prolonged hospitalization warrants consideration. Patients with established CAD presenting with acute pulmonary edema are likely to have preexisting, guideline-directed pharmacological management including β-blockers, statins, and renin-angiotensin system inhibitors. This established therapeutic framework, combined with prior specialist contact, may facilitate more structured inpatient management and earlier discharge decisions. An alternative explanation is that, in a cohort where pulmonary edema arises from decompensated chronic heart failure rather than an acute ischemic event, patients with known CAD may represent a more hemodynamically stable phenotype in whom the acute episode resolves more predictably. This interpretation remains speculative given the observational design and the absence of data on specific in hospital therapies, and merits prospective evaluation. The broader interaction between CAD, comorbidity burden, and frailty in heart failure – and how this interaction shapes clinical outcomes – has been outlined in the Heart Failure Association of the European Society of Cardiology position paper on frailty in patients with heart failure.^[14]^ These findings are consistent with evidence from acute illness settings showing that frailty instruments may have limited discriminative accuracy when applied during acute physiological decompensation.^[20,21]^ Therefore, even if bedside frailty instruments do not detect an independent short-term effect, clinically relevant stratification may still be achieved through the comorbidity burden analysis.

Frailty may predict short-term mortality in AHF; this association has not been observed in all cohorts. In 1 study, frailty independently predicted 30-day mortality, underscoring its potential prognostic relevance in selected populations.^[22]^ The literature also includes studies in which frailty did not predict short-term mortality, consistent with our findings. For example, a study of 100 older patients hospitalized for AHF found that frailty, measured using the CFS, was associated with 1-year mortality but not in-hospital mortality.^[23]^

In our study, several factors might have masked the effect of frailty on mortality and length of stay. First, the effect of frailty may not appear as an independent determinant of in-hospital mortality during the acute inpatient phase, when illness severity and complications tend to dominate. Instead, the impact more commonly emerges after discharge, manifesting as adverse outcomes such as functional deterioration, institutionalization, and recurrent healthcare utilization. This pattern can be explained by a “time-horizon mismatch.” This interpretation is also consistent with evidence from large systematic reviews and meta-analyses of HF, which show that frailty is associated with a greater hospitalization burden (e.g., longer length of stay and more frequent prior HF admissions) and worse long-term post-discharge outcomes (including mortality, readmission, functional decline, and increased care needs).^[24]^

Frailty was assessed at the time of emergency department presentation in this study, based on each patient’s clinical status at evaluation rather than their premorbid functional baseline. This approach carries a specific risk of misclassification: acute physiological derangement associated with pulmonary edema – including dyspnea at rest, severe fatigue, and reduced mobility – may transiently worsen performance on function-based instruments, leading to overestimation of frailty severity in a subset of patients. The CFS, for instance, is intended to reflect the patient’s habitual functional status rather than their condition at the peak of an acute illness. In 1 study of hospitalized older adults, frailty index scores increased at the time of hospitalization compared with baseline in a substantial proportion of patients, with partial recovery observed after discharge in a subset.^[22]^ In the short term, frailty can be both biologically and clinically dynamic; a longitudinal study showed that short-term fluctuations in the frailty index were not random and became particularly pronounced around health events such as hospitalization, implying that measurements repeated over weeks may be more variable in older adults with more severe illness.^[25]^ When applied at presentation, frailty instruments may therefore capture acute decompensation rather than true baseline vulnerability, reducing their discriminative value for outcomes that depend on underlying biological frailty. This misclassification is expected to attenuate the observed association between frailty and short-term outcomes, biasing results toward the null.

Third, frailty instruments in our study classified a substantial proportion of patients as “frail,” which can limit their ability to discriminate. This can create a ceiling effect, making it difficult to capture outcome gradients that reflect increasing risk levels. Even when a measurement tool is valid and reliable, its discriminative ability may diminish, and the overall model performance may decline if most patients in the cohort are already grouped within a high-risk/frail tier.

In the heart failure literature, frailty is a clinically meaningful prognostic marker in the post-discharge period. In a large prospective study, the prevalence of baseline frailty in patients hospitalized for AHF was 71%, and frailty independently predicted 12-month mortality; the 30-day readmission rate in that cohort was 24%.^[26]^ In a longitudinal HF cohort using the TFI, frailty was associated with higher rates of unplanned readmissions and death during short post-discharge follow-up windows at 30, 60, and 90 days.^[27]^ In a prospective emergency department cohort, tools such as the CFS and PRISMA-7 were used to assess outcomes more sensitive to frailty, such as functional decline and nursing home placement, at 30 days and 6 months.^[28]^ As the literature suggests that the impact of frailty in HF often becomes more evident during the post-discharge period, frailty assessment in patients hospitalized with AHF may help anticipate adverse outcomes in the medium-to long-term. Therefore, in routine acute care, frailty screening in patients with AHF may be more appropriately used for care planning than as a stand-alone, short-term prognostic instrument.

The present study also contributes to a growing body of evidence highlighting the limitations of conventional prognostic tools when applied to older adults with acute cardiac conditions. Standard risk prediction models were largely developed and validated in younger, less comorbid populations, and their discriminative accuracy may be reduced in elderly patients with complex multimorbidity profiles. Recent work has demonstrated this limitation across several clinical contexts: dedicated models for myocardial injury prediction in elderly patients undergoing nonelective surgery,^[29]^ comparative analyses of mortality prediction scores in elderly patients with implantable cardioverter-defibrillators for heart failure with reduced ejection fraction,^[28]^ and investigations into the prognostic impact of comorbidities such as atrial fibrillation in elderly patients undergoing high-risk procedures^[30]^ have collectively pointed to the need for age-adapted, population-specific risk assessment frameworks. The findingsof the present study align with this pattern: even well-validated frailty instruments showed limited prognostic precision when applied to acutely ill older adults, suggesting that short-term outcome prediction in this population may require multidimensional approaches that integrate acute illness severity, comorbidity burden, and functional status rather than relying on a single screening tool.

There are many limitations to this research that should be acknowledged. The fact that the study was conducted at 1 center (a large city-based university hospital) limits how widely applicable the results can be. When frailty is measured when a person presents to an Emergency Department it does not always represent their pre-illness functional ability as acute physiological abnormalities can cause temporary worsening of scores on function-based assessments thereby introducing a potential source of bias towards the null association. Due to the requirement for patients’ active participation in assessing frailty, those requiring endotracheal intubation or having altered mental status were excluded from participating in the study and thus likely underrepresented those who would have been classified as the most severely ill. Acute illness severity data such as natriuretic peptide levels, ICU admission rates and noninvasive ventilation requirements were also missing, which limited the number of confounders that could be adjusted for in the multivariate models. Additionally, no sample size calculation had been previously completed; therefore, there were 17 in-hospital deaths which produced very wide CIs and imprecise estimates of mortality. Lastly, both self-report and clinician administered frailty tools were used while other laboratory and performance-based frailty measurement tools were not utilized resulting in a limitation that prevents comparison of measurements across different frailty definitions.

Frailty was highly prevalent in older patients hospitalized with acute pulmonary edema, affecting approximately 71% to 73% of participants across all 3 instruments; however, neither one of them demonstrated statistically significant predictive value for either mortality while hospitalized or longer length of stay after controlling for potential covariates. The very poor discrimination of frailty measurements for 2 studied outcomes (as evidenced by AUC values close to 0.5) suggests that measuring frailty during an acute episode of decompensation will not provide an accurate measure of the biological susceptibility underlying an individual’s short-term inpatient outcomes. Therefore, assessing frailty in the emergency setting may be more useful for supporting care decisions and assessing post-discharge risk factors than as a separate short-term predictor of inpatient outcomes. Larger multicenter studies are required to determine whether frailty predicts in-hospital mortality/length-of-stay in this population utilizing a pre-illness assessment of frailty and objective markers of illness severity.

## Acknowledgments

The authors used Paperpal (an AI-based writing assistant) for English language editing and proofreading to improve the clarity and grammatical accuracy of the manuscript. After the automated suggestions were applied, the authors reviewed and edited the content to ensure technical accuracy and take full responsibility for the final version.

## Author contributions

**Conceptualization:** Derya Dilaver, Görkem Alper Solakoğlu.

**Data curation:** Derya Dilaver, Halil Emre Bilgiç, Uğur Durmuş.

**Formal analysis:** Derya Dilaver, Görkem Alper Solakoğlu.

**Investigation:** Derya Dilaver, Görkem Alper Solakoğlu, Halil Emre Bilgiç, Uğur Durmuş.

**Methodology:** Derya Dilaver, Görkem Alper Solakoğlu, Halil Emre Bilgiç.

**Project administration:** Derya Dilaver.

**Resources:** Derya Dilaver, Halil Emre Bilgiç.

**Supervision:** Görkem Alper Solakoğlu.

**Validation:** Derya Dilaver, Görkem Alper Solakoğlu, Uğur Durmuş.

**Writing – original draft:** Görkem Alper Solakoğlu.

**Writing – review & editing:** Derya Dilaver, Görkem Alper Solakoğlu, Halil Emre Bilgiç, Uğur Durmuş.
